# CCR3 Expression in Relation to Delayed Microbleeds in a Rat Model of Large Vessel Occlusion

**DOI:** 10.33696/neurol.5.082

**Published:** 2024

**Authors:** Sydney M Claypoole, Jacqueline A Frank, Sarah J Messmer, Keith R Pennypacker

**Affiliations:** 1 Department of Neurology, University of Kentucky, Lexington, KY 40536, USA; 2 Department of Neurosurgery, University of Kentucky, Lexington, KY 40536, USA; 3 Department of Neuroscience, University of Kentucky, Lexington, KY 40536, USA

**Keywords:** Chemokine, Endothelium, Molecular stroke research, Neuroimmune, Neuroinflammation

## Abstract

Thirty percent of ischemic stroke patients develop vascular cognitive impairment and dementia (VCID) within 1 year of stroke onset. The expression of C-C motif chemokine receptor 3 (CCR3) is associated with endothelial dysfunction and memory impairment. CCR3 has been reported to increase after experimental stroke and in human stroke patients. Using an *in vivo* model of stroke, our study aims to link CCR3 expression with endothelial dysfunction in this rodent stroke model.

**Methods:**

5-hour transient Middle Cerebral Artery Occlusion (5t-MCAO) or sham surgery was performed on rats and tissue collected at 3- and 30-days post-stroke. We measured the change in expression of CCR3 and its ligands in the venous blood before and after occlusion in the rat model.

Immunohistochemistry was performed on consecutive coronal brain sections using Prussian blue to visualize microbleeds and DAB to visualize CCR3. Images were quantified using HALO.

**Results:**

Using linear regression, we found that increased expression of CCR3 and its ligands after stroke were positively correlated with infarct volume. CCR3 expression was significantly increased in the ipsilateral hemisphere at 30 days post 5t-MCAO. Prussian blue staining was significantly increased in ipsilateral sections at 30 days post-stroke. Immunostaining for CCR3 was primarily detected in endothelium in areas of Prussian blue staining.

**Conclusions:**

Our results demonstrate that CCR3 expression is associated with the presence of microbleeds at 30 days but not 3 days post-stroke in the ipsilateral hemisphere, and further supports the link between CCR3 and the endothelial dysfunction that is associated with VCID. CCR3 and its inflammatory pathway is a potential target for reducing endothelial dysfunction after ischemic stroke that may lead to VCID.

## Introduction

Ischemic stroke is a leading cause of morbidity and mortality throughout the world [1]. Approximately 30% of all stroke patients develop vascular cognitive impairment and dementia (VCID) within 1 year of stroke onset [2]. Endothelial dysfunction with subsequent microbleeds have been known to be associated with VCID [3]. However, the exact molecular mechanisms of stroke induced VCID are unknown. After an ischemic insult has occurred, the brain attempts to repair its vascular system and restore circulation to the affected area [4]. This process is called neovascularization [5]. While this process is presumed to be beneficial following stroke, the formation of new vessels can be overstimulated, leading to aberrant growth and endothelial dysfunction. These result in endothelial permeability and microbleeds, which subsequently are related to the development of VCID [3].

The expression of C-C motif chemokine receptor 3 (CCR3) has been reported to increase after experimental stroke [6], and the inhibition of CCR3 has been demonstrated as a protective factor for neurons after an ischemic event [7]. Our group previously reported that CCR3 expression is significantly associated with infarct volume and edema in stroke patients undergoing thrombectomy [8]. We have determined that CCR3 expression is increased in venous blood in our rat stroke model and patients undergoing thrombectomy [9]. The expression of ligands for CCR3, CCL5, CCL11 and CCL24, have been studied in stroke models. CCL5 knock-out mice have been reported to have smaller infarct volumes [10], while CCL11 is associated with neuronal death and predicts long-term disability [11,12]. CCL24 was reported to be significantly increased after symptomatic intracranial hemorrhage following ischemic stroke, a rare but severe complication of intravenous thrombolysis, suggesting its role as a potential biomarker for hemorrhagic transformation [13].

CCR3 and its ligands have been implicated in the process of endothelial dysfunction in a variety of diseases. In atherosclerosis, activation of the CCR3 receptor resulted in an increase of permeability in coronary arteries [14]. Further, the CCR3 molecular pathway is a Th2 immune response, reported to promote angiogenesis and increase angiogenic factors by increasing the expression of VEGF [15]. Inhibiting CCR3 has demonstrated efficacy in reducing Th2 immune responses for treatment of severe asthma [16,17], and our group found the Th2 immune response itself to be positively associated with increased infarct volume and edema in stroke patients undergoing thrombectomy [8]. In aging macular degeneration (AMD), overstimulation of new vessel formation can lead to dysfunction, endothelial permeability, and microbleeds [18–20], similarly to VCID. Thus, CCR3 signaling has already been identified as a therapeutic target for AMD treatment [21,22]. Novel CCR3 antagonists have been developed to treat AMD and exhibited success in inhibiting vascular permeability and neovascularization in the eye [23,24]. A CCR3 antagonist, GW766994X, has been shown to reduce endothelial permeability [24], as well as factors that impair cognition with no adverse effects displayed in a human clinical trial [25].

Advances in treatment for stroke patients, such as intravenous t-PA and mechanical thrombectomy, have significantly improved the ability to restore blood flow during stroke and improve outcomes for some patients up to 24 hours after stroke onset [26,27]. However, despite these effective strategies to mitigate the immediate effects of stroke, patients still undergo significant long-term cognitive and functional effects from the injury [28], including VCID [2].

Therefore, there remains a clinical need for adjuvant therapies to maximize neurological and cognitive outcomes. Animal models for VCID are needed to identify potential therapeutic targets for treatment. This study aims to show that CCR3 expression in this rodent model that mimics thrombectomy is associated with endothelial dysfunction that is a characteristic of VCID.

## Methods

### Middle cerebral artery occlusion (MCAO) with reperfusion (5t-MCAO)

Sprague Dawley rats (18 months old) underwent a sham or 5-hour middle cerebral artery occlusion (5t-MCAO) and were then euthanized 3- or 30-days post stroke. Our 5t-MCAO procedure that we used has previously been published [9]. In summary, animals were placed in an induction chamber and anesthetized with 5% isoflurane/oxygen. Anesthesia was maintained with a constant flow of 2–3% isoflurane in 100% oxygen at a rate of 1 L/min. After a midline vertical neck incision was made, the common (CCA) external (ECA), and internal (ICA) systems were dissected and isolated. The ECA was isolated and used to access the arterial system. A 30- mm 4–0 or 5–0 monofilament dependent on the animal’s weight was fed distally into the ECA and advanced through the system to the ICA until the filament reached the MCA. The animal was allowed to gain consciousness for 5 hours and was then re-anesthetized for removal of the filament.

To minimize the potential for experimenter bias, which has been identified in the past as a major contributor to past clinical failures [29], all rodent studies were performed with blinding.

Randomization was also conducted whenever applicable, using a computerized random number generator to determine competitive treatments (stroke vs. sham groups). Male and female rodents were numbered sequentially starting with the first cage and randomly assigned to surgery days/times to avoid experiments on all animals of one sex being performed on a single day. We evaluated both sexes, but only in aged cohorts, to match the average age of clinical stroke patients undergoing thrombectomy. This experimental approach using aged rats of both sexes abides by the guidelines and standards outline in the Stroke Therapy Academic Industry Protocols (STAIR) criteria [30].

### Rodent magnetic resonance imaging

MRI images were acquired on a 7T Bruker Biospin horizontal bore system (7.0T, 30cm, 300Hz) equipped with a triple-axis gradient system (630 mT/m and 6300 T/m/s) with a closed cycle. T2- weighted, DTI (Diffusion tensor imaging), and ASL (Arterial spin labeling) MRI perfusion imaging were performed. Edema and infarct volumes were determined from the AD volumes and the T2-weighted MRI images by manual segmentation using ITK-SNAP [31]. pCASL image analysis was employed with in-house written codes in MATLAB (MathWorks, Natick, MA) to obtain quantitative cerebral blood flow (CBF) [32,33]. Rats were anesthetized with an average of 2.25% isoflurane in oxygen, while female rats were anesthetized with an average of 1.75% isoflurane in oxygen using MRI-compatible CWE Inc. equipment (Ardmore, PA). The animals were held in place on a Bruker scanning bed with a tooth bar and tape. Body temperature, heart rate, and respiratory rate were continuously monitored throughout the MRI scans (SA Instruments, Inc., Stony Brook, NY). The animals were maintained at 37°C with a water heating system built into the scanning bed. The scanning procedure took approximately 60 minutes per animal. The perfusion (CBF), white matter hypersensitivity (WMH), DTI, and T2-weighted MRI images were analyzed by a blinded neuroradiologist to measure the infarct volume and edema volume. These volumes were counted, and the numbers were normalized to the number of images counted to provide a per-section count. The volume of brain parenchyma demonstrating restricted diffusion (infarct volume) visibly affected by cerebral edema (edema volume) was calculated by manual segmentation using ITK-SNAP software. The volume of brain parenchyma visibly affected by cerebral edema (edema volume) was calculated in a similar fashion.

### RNA isolation and quantification

The methods employed for RNA extraction and amplification were adapted from Martha et al. [8]. Blood was collected from the jugular vein at three different time points: immediately prior to MCAO surgery, 5 minutes after MCA reperfusion, and 72 hours post-MCAO procedure. Sham rats underwent the complete 5t-MCAO procedure, with the exception that the filament was not placed to induce a stroke; blood samples were taken at the same time points for sham animals. Total RNA was extracted from the pellet/buffy coat using the Nucleospin Blood Kit (Macherey-Nagel, Düren, Germany). RNA quantity was assessed with a Nanodrop Lite (Thermo-Fisher; Waltham, MA). cDNA synthesis utilized the RT^2^ First Strand Kit from Qiagen, and gene expression analysis of 84 genes was performed using the ABI StepOne Plus Qiagen (Germantown, MD) and the RT^2^ Profiler Rat Chemokine and Receptor Array from Qiagen.

### Determination of endothelial permeability

Frozen coronal brain sections containing the infarct were immunostained for expression of CCR3 (Novus (NBP1–77065)-Rabbit). Images were taken using an Axio Scan Z1 slide scanner and quantified using HALO software version 2.1 (v1.0.20.1). Quantification was expressed as percent area within the striatal penumbra consistent between rats.

Additional frozen coronal brain sections (1.7 to −3.3 mm from Bregma) were stained with Prussian blue (kit ab150674, Abcam) to visualize microbleeds and quantified using HALO. These specific points have been used in past publications by our lab to quantify infarct and edema volumes in rat brains after experimental stroke [9]. Sections were incubated in an iron stain followed by nuclear fast red. A blue coloration occurred around the vessels that were permeable. We focused on Prussian blue dye-stained areas outside of the infarct as demarcated by intact brain parenchyma.

In order to determine if CCR3-expressing endothelial cells were involved in the leakage of blood vessels, co-staining was performed on frozen coronal brain sections that were previously stained with Prussian blue dye. Rabbit antibodies (CCR3: Novus (NBP1–77065)-Rabbit; vWF: Abcam (ab6994)-Rabbit) for CCR3 and Von Willebrand Factor (vWF), an endothelial cell marker, were used to identify overlapping expression of these two proteins. Immediately adjacent, consecutive coronal brain sections from the same rat were immunostained with CCR3 and vWF, respectively. Both stains were antibody stains and thus sections were processed separately. The Prussian blue staining protocol was then followed as above. CCR3 DAB staining was performed on the same 30-day post stroke rat.

## Results

### Cytokines predict infarct volume in rats after 5t-MCAO

**Figure 1.**
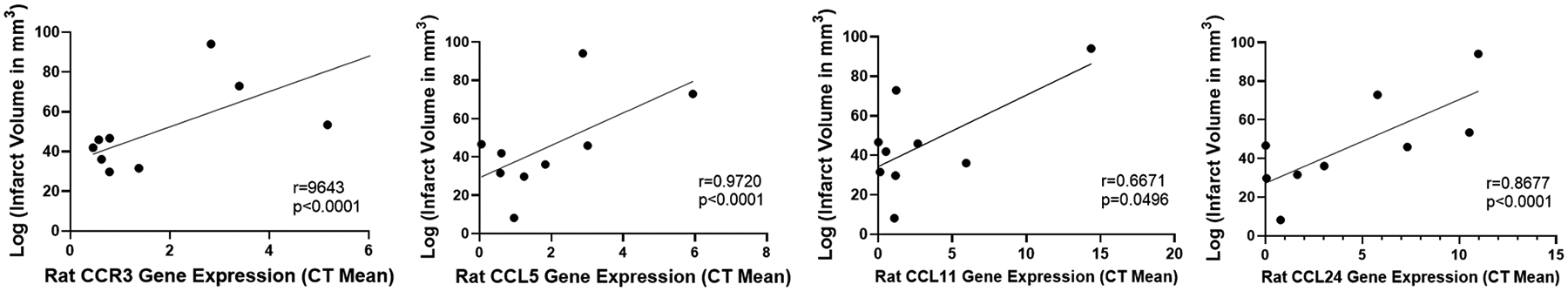
CCR3, CCL11, CCL24, and CCL5 c hange in gene expression are positively correlated with infarct volume in rats following ischemic stroke (n=9).

We measured the change in expression of CCR3 and its ligands (CCL5, CCL11, and CCL24) in the venous blood prior to and after occlusion in our transient rat model of ischemic stroke. Using linear regression, we found that increased expression of these proteins after stroke positively correlated with infarct volume (Figure 1). Our group has previously reported that, when using machine learning analysis, CCR3 expression was a predictor of both infarct and edema volumes, while CCL11 was a predictor of edema volume from the blood of human stroke patients [8].

Expression of CCR3 signaling genes in our rat model of thrombectomy reflects a similar pattern to the data retrieved from human stroke patients, indicating that the expression of these genes is associated with increased stroke-induced damage to the brain.

### CCR3, vWF, and Prussian blue staining 30 days following 5t-MCAO in rats showed increased CCR3 expression in areas of microbleeds

**Figure 2.**
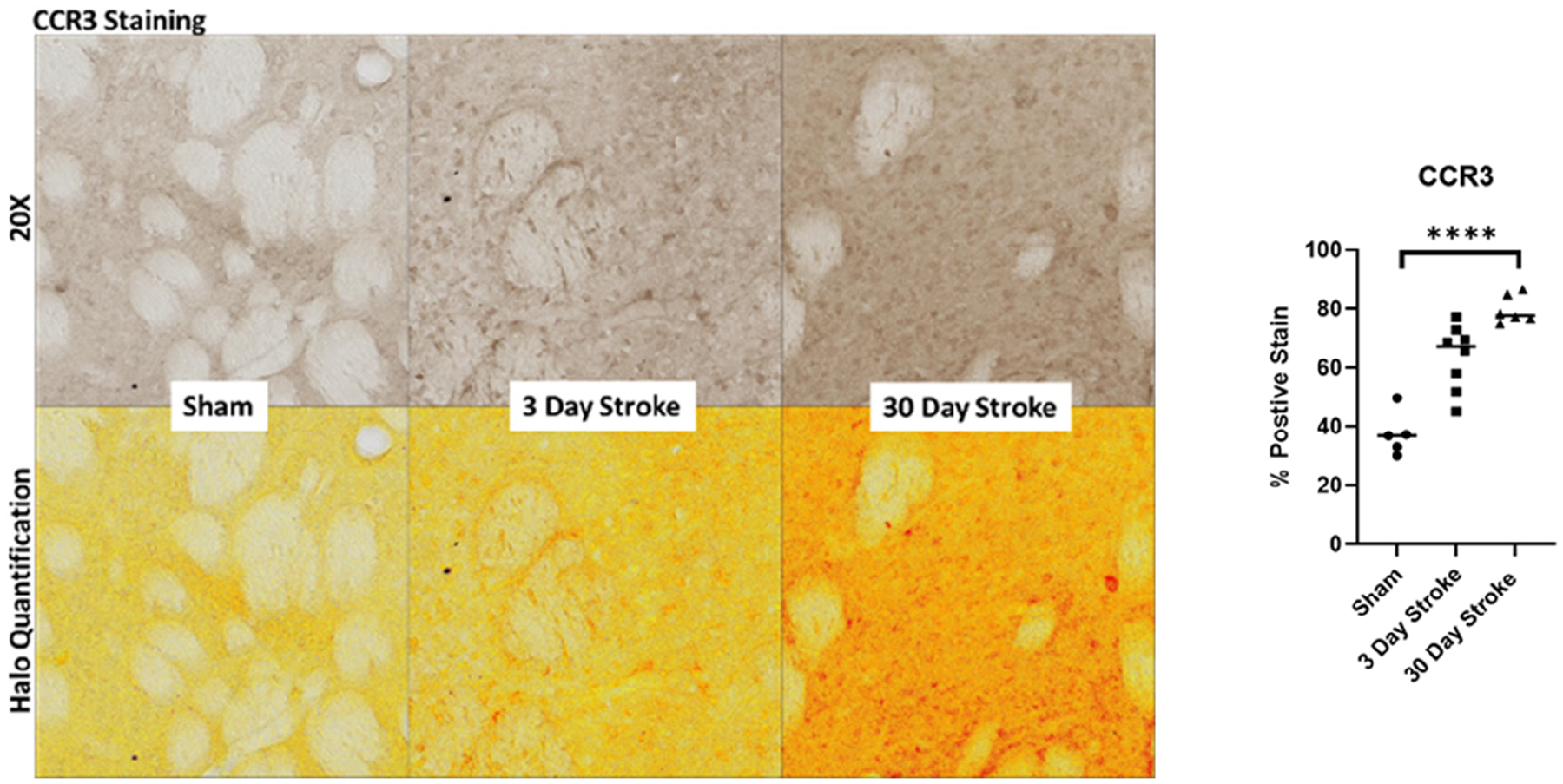
**(a)** CC R3 immunostaining with HALO quan tification expression after 5t-MCAO at 3 and 30 days. **(b)** analysis by ANOVA. ****p<0.001.

**Figure 3.**
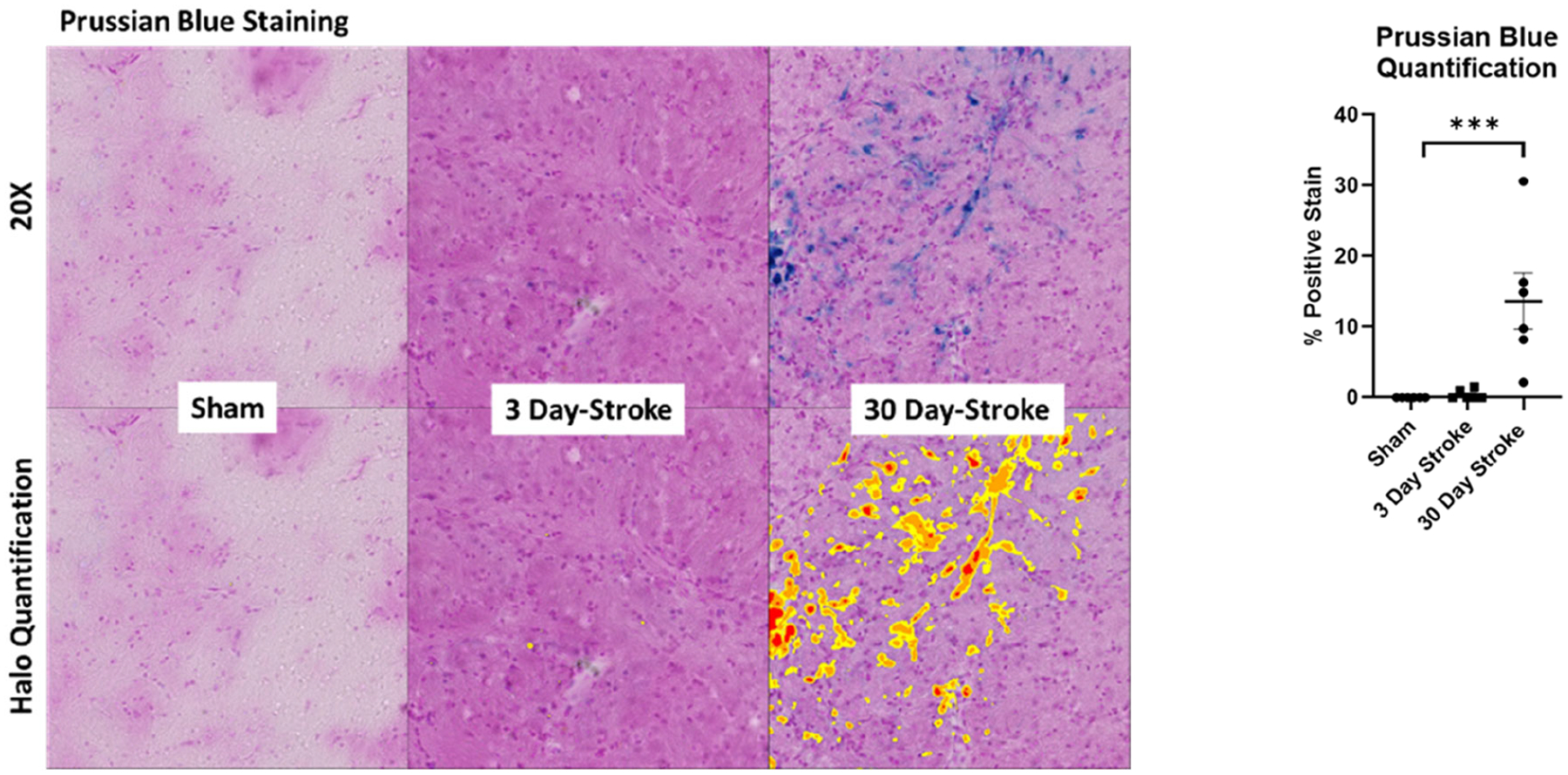
**(a)** Prussian blue staining following sham operated or 5t-MCAO at 3- and 30- days post-stroke. **(b)** HALO quantification ANOVA ***p<0.001.

Brain sections containing the infarct were immunostained for expression of CCR3 (Figure 2a). CCR3 expression staining significantly increased following stroke at 30 days (p <0.001; Figure 2b). Sections stained with Prussian blue (Figure 3a) demonstrated a significant increase following stroke at 30 days as analyzed with ANOVA (Figure 3b). Together, these results indicate microbleeds residing outside the infarct area, an outcome that is similarly associated with the pathogenesis of VCID.

**Figure 4.**
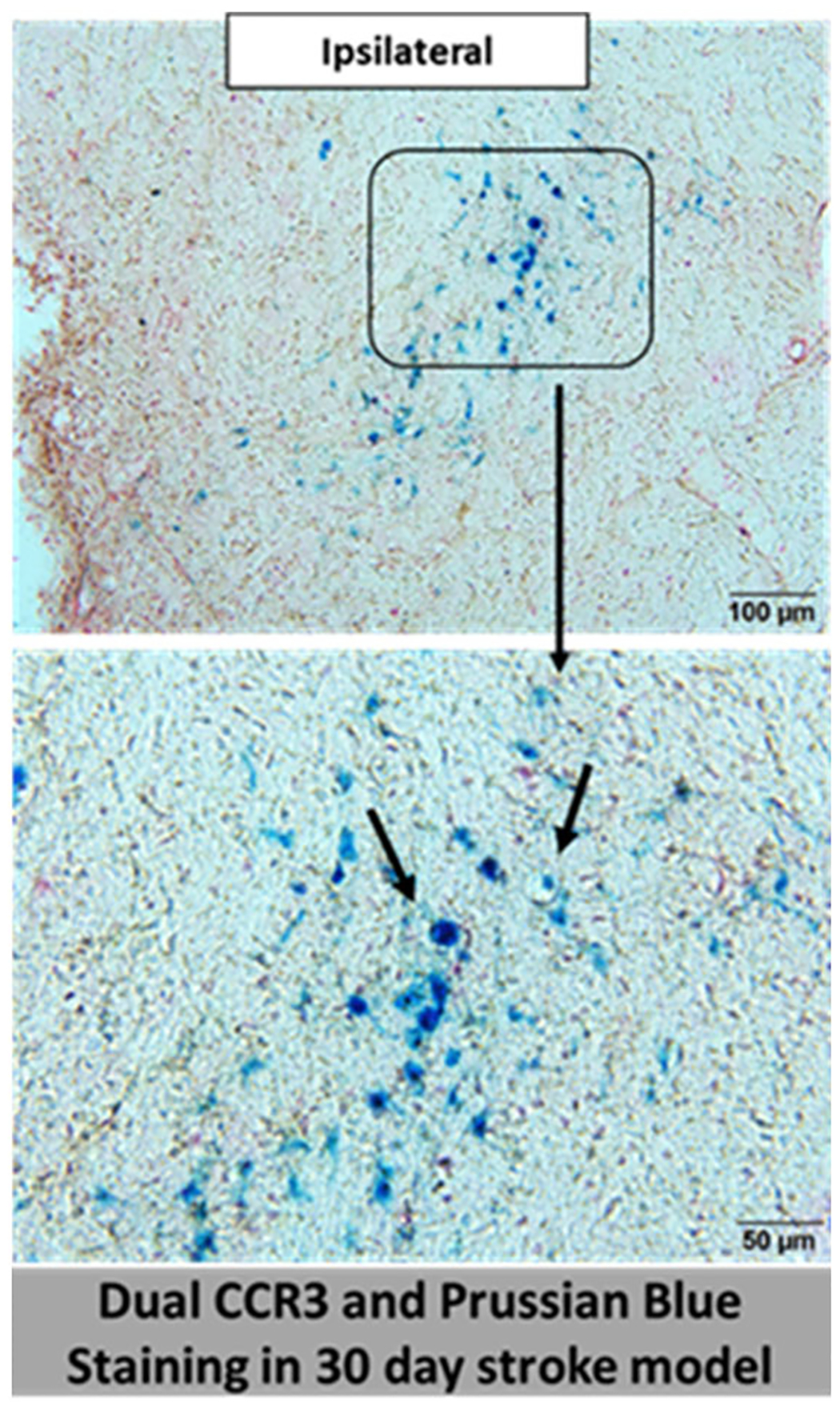
Dual Prussian blue and CCR3 DAB staining in 30-day post 5t-MCAO.

**Figure 5.**
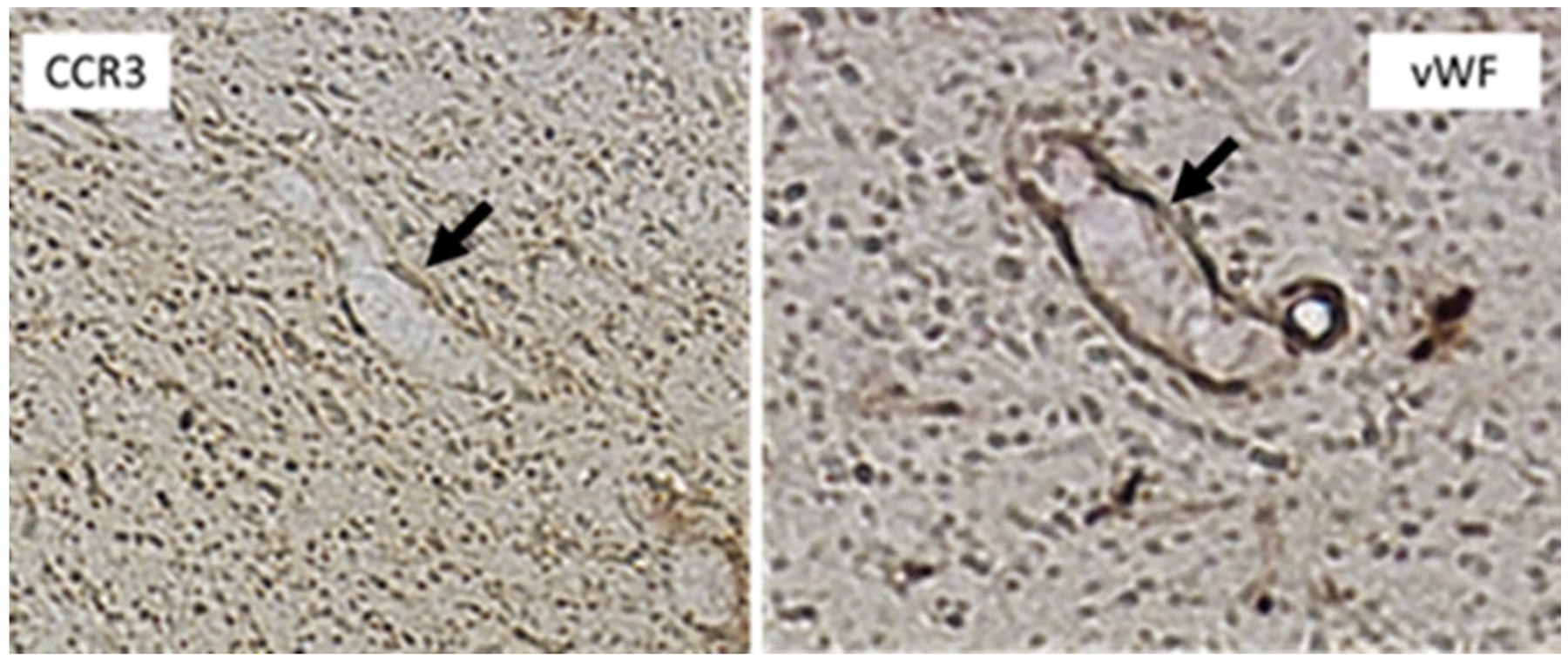
Expression of CCR3 and Von Willebrand Factor (VWF), an endothelial cell marker, in a blood vessel (arrows) in consecutive brain sections from the same rat.

Furthermore, immunostaining for CCR3 was readily detected in areas that were labeled with Prussian Blue, suggesting that CCR3-expressing endothelial cells are involved in the leakage of blood vessels (Figure 4). Likewise, we stained consecutive brain sections with CCR3 and vWF antibodies to identify overlapping expression between these two proteins. Figure 5 depicts expression of CCR3 and vWF in a blood vessel (arrows) in consecutive brain sections from the same rat.

## Discussion

As previously mentioned, 30% of stroke patients develop vascular cognitive impairment and dementia (VCID) within 1 year of their stroke [2]. This burden of morbidity on patients, as well as a general lack of understanding of the underlying mechanism, necessitates the development of an animal model of this disease process. Previously, we have reported that CCR3 is a significant predictor for both infarct volume and edema in stroke patients undergoing thrombectomy. Our results revealed promising support of an animal model that CCR3 signaling is related to endothelial dysfunction. We found that the change in gene expression of CCR3 and ligands (CCL5, CCL11, and CCL24) is positively correlated with infarct volume in our transient rat model of ischemic stroke. CCR3 and Prussian blue staining significantly increased in rat brain sections following stroke at 30 days, but not at 3 days, and co-staining with CCR3 and vWF antibodies demonstrated overlapping expression. These findings suggest the presence of microbleeds and propose that CCR3-expressing endothelial cells were involved with microvessel leakage and ensuing injury.

Endothelial dysfunction and subsequent microbleeds have been cited as a mechanism of VCID secondary to aberrant neovascularization mediated by proangiogenic factors [3]. In particular, microbleeds residing outside of the infarct area are associated with VCID pathogenesis, Microbleeds found in rat brains after the stroke procedure in this study are associated with increased CCR3 expression.

CCR3 is involved in several other diseases in which endothelial dysfunction is associated with pathogenesis, and it has been successfully targeted as a treatment for these conditions.

CCR3 receptor activation in atherosclerosis increases endothelial permeability in the coronary arteries [14] and pharmaceutical companies have targeted CCR3 to block the Th2 immune response for treatment of severe asthma [16,17]. Most notably, in aging macular degeneration (AMD), new vessel formation is overstimulated leading to dysfunctional endothelium, vessel permeability, and microbleeds [18–20]. In addition, CCR3 ligand CCL24 is a biomarker for AMD [23] and could be similarly explored as a biomarker for VCID and other stroke outcomes. The aberrant AMD growth is associated with CCR3 and its ligands and is a current therapeutic target for its treatment [21,22]. Novel CCR3 antagonists, such as GW766994X, have demonstrated efficacy in the inhibition of neovascularization and vascular permeability in the eye [23,24]. No adverse effects of GW766994X were reported in a human clinical trial [25]. Mice deficient in CCR3 exhibited significantly reduced Aβ production and subsequent processes that are associated with deficient learning and memory performance in a model of age- related dementia [34]. The expression of CCR3 has been linked to age related dementia [36], and CCR3 antagonists have been shown to enhance cognitive performance in rodents [35]. Increased expression of CCR3 and its ligands (CCL5, CCL11, and CCL24) are also associated with cognitive decline associated with aging [36–41]. Thus, CCR3 antagonists are a logical next step to investigate the vascular permeability that leads to cognitive impairment in future animal models of stroke.

In stroke research, CCR3 has been implicated as a mediator of neuronal injury after ischemic insult [7]. It is related to the Th2 immune response, which is a T-cell response largely associated with autoimmunity, allergy, and cancer [42,43]. One study demonstrated a systemic shift by the immune system toward Th2 response in the late post-acute phase of stroke [44]. Th2 cells express CCR3, which acts to bind chemokines and direct their migration towards the site of injury or infection [45,46]. Our group previously employed machine learning to predict infarct and edema volumes after acute ischemic stroke [8]. Th2-related genes, particularly CCR3, in arterial blood samples were indicative of infarct volume and edema. The study identified an association between CCR3, related genes, and type 2 diabetes mellitus as predictors for stroke outcomes. Further investigation is needed to examine the relationship between CCR3 and infarct and edema volumes after ischemic stroke in both animal models and human patients. Inhibiting CCR3 may also have additional beneficial effects after stroke by diminishing the Th2 response.

## Conclusions

Despite the development of effective strategies to restore blood flow and improve outcomes after ischemic events, such as t-PA and mechanical thrombectomy, patients still endure significant long-term cognitive and functional outcomes from the injury. There remains a clinical need for adjuvant therapies to maximize neurological outcomes. This present study offers insight into a molecular signaling pathway that appears to play a role in endothelial permeability after ischemic stroke that has been associated with VCID. CCR3 signaling has previously been associated with endothelial permeability in AMD, but not in stroke. Our results demonstrate that CCR3 expression is increased 30 days after stroke in areas of microbleeds in our animal model that mimics patients undergoing thrombectomy. These data further support the link between CCR3 and its inflammatory pathway with the endothelial dysfunction after stroke. Thus, future studies should continue to determine the impact of CCR3 signaling on cognitive and functional outcomes after stroke to determine if it is a therapeutic target to reduce stroke induced detrimental effects.
